# Comparative Characterization and Pathogenicity of a Novel Porcine Epidemic Diarrhea Virus (PEDV) with a Naturally Occurring Truncated ORF3 Gene Coinfected with PEDVs Possessing an Intact ORF3 Gene in Piglets

**DOI:** 10.3390/v13081562

**Published:** 2021-08-07

**Authors:** Ying Lu, Weijian Huang, Lian Zhong, Yibin Qin, Xueting Liu, Chunjie Yang, Ruomu Wang, Xueli Su, Chen Du, Xue Mi, Hejie Wang, Ying He, Wu Zhao, Ying Chen, Zuzhang Wei, Kang Ouyang

**Affiliations:** 1 College of Animal Science and Technology, Guangxi University, Nanning 530005, China; luying9986@163.com (Y.L.); huangweijian-1@163.com (W.H.); zhonglianzl20@163.com (L.Z.); yangyuanqiu2090@163.com (X.L.); 18317715002@sina.cn (C.Y.); wangruomu2021@163.com (R.W.); suxueli@aliyun.com (X.S.); dchenj01@163.com (C.D.); mx506831280@sina.com (X.M.); whj5355006@163.com (H.W.); yingchen@gxu.edu.cn (Y.C.); Zuzhangwei@gxu.edu.cn (Z.W.); 2 Guangxi Veterinary Research Institute, Nanning 530005, China; qinyibin5188@163.com (Y.Q.); yinghe2@gmail.com (Y.H.); zhaowu168866@163.com (W.Z.)

**Keywords:** porcine epidemic diarrhea virus, coinfection, ORF3, molecular characteristics, pathogenicity, virulence

## Abstract

Coinfection caused by various genotypes of porcine epidemic diarrhea virus (PEDV) is a new disease situation. We previously reported the coexistence of PEDV strains containing different ORF3 genotypes in China. In this study, the PEDV strains 17GXCZ-1ORF3d and 17GXCZ-1ORF3c were isolated and plaque-purified from the same piglet, which had a natural large deletion at the 172–554 bp position of the ORF3 gene or possessed a complete ORF3 gene, respectively. Meanwhile, 17GXCZ-1ORF3d had >99% nt identity with 17GXCZ-1ORF3c in the 5′UTR, ORF1a/1b, S, E, M, N and 3′UTR regions but only demonstrated low nucleotide identities (80.5%) in the ORF3 gene. To elucidate the pathogenicity, 7-day-old piglets were infected. Piglets infected with these two PEDV strains exhibited severe clinical signs and shed the virus at the highest level within 96 hpi. Compared with the piglets inoculated with the 17GXCZ-1ORF3c strain, the piglets inoculated with the 17GXCZ-1ORF3d strain had higher mortality rates (75% vs. 50%), an earlier onset of clinical signs with a significantly higher diarrhea score, lower VH:CD ratios and a higher percentage of PEDV-positive enterocytes. This study is the first to report PEDV coinfections with different ORF3 genotypes, and a PEDV strain with a large deletion in the ORF3 gene might have the advantage of a potential genetic marker, which would be useful during vaccine development.

## 1. Introduction

Porcine epidemic diarrhea (PED), characterized by severe acute watery diarrhea, vomiting, dehydration and growth retardation in pigs of all ages, especially newborn piglets, causes tremendous economic losses to the swine industry worldwide [1,2,3]. PED virus (PEDV) was first reported in Europe in the late 1970s, and the virus subsequently appeared in China in the 1980s [4]. At the end of 2010, a highly virulent PEDV variant was observed in China and resulted in a massive mortality rate in piglets [5]. Subsequently, the outbreak of a newly emerged US strain occurred in the United States in 2013 [6], and there has been a spread of PEDV strains to other swine producing regions and countries such as Taiwan, Japan and Italy [7,8].

PEDV is an enveloped, single-stranded, positive-sense RNA virus belonging to the family Coronaviridae [9]. The viral genome is about 28 kb in length and includes seven open reading frames (ORFs), encoding two nonstructural proteins (ORF1a and ORF1b), a spike(S) protein, an accessory protein (ORF3), an envelope(E) protein as well as membrane (M) and nucleocapsid (N) proteins. The S glycoprotein plays a pivotal role in viral entry [10,11] and is significant for its genetic relatedness and epidemiological status of PEDV [12].

The PEDVs are comprised of genotypes 1 (G1) and 2 (G2) based on the S gene, and G2 has been further classified into G2a and G2b subtypes [13]. Apart from the S gene, the ORF3 protein exhibits a genetic variation among PEDVs [14] and is a valuable tool for the molecular epidemiologic studies of PEDV infections. The PEDV ORF3 is a single accessory protein gene that is genus-specific for coronaviruses [15]. It is located between the S and E genes, encodes an ion channel protein of 224 amino acids (aa) [16] and interacts with the S protein, which correlates with the ability of the protein to regulate or interfere with virus replication [14]. The truncated ORF3 is mostly found in cell adapted or attenuated PEDV [17,18].

Different enteric viruses commonly cause coinfections with PEDVs [19], but in recent years, coinfection caused by the various genotypes of PEDV has appeared as a new disease situation. The coinfections caused by the two S gene genotypes of PEDV were first reported in Japan in 2017 with a frequency rate of 32.7% [20]. New tropisms of PEDV in piglets naturally coinfected by S1 NTD-del PEDV variants and S-intact PEDVs were identified [21]. Viral replication is normally enhanced during the coinfection of the two S genotypes of PEDV in piglets, and the clinical symptoms caused by the co-infection were as severe as those of the highly virulent PEDV alone [22,23]. However, coinfections caused by different ORF3 genotypes of PEDV have not been reported.

PEDV ORF3 deletion strains have been reported in China [24]. In our previous work, we found that two ORF3 genotypes were present in the same piglet, one of which had a naturally truncated ORF3 gene that contained a continuous 382 nucleotides deletion from 172–554 bp, and the other of which had an intact ORF3 gene of 675 nucleotides [25]. Thus, we suspected that a co-infection with two different PEDV strains occurred in the piglet. Nevertheless, the comparative pathogenesis of PEDV with different ORF3 genotypes has not been investigated yet. In this study, two purified PEDV strains with different ORF3 genotypes were obtained from the same piglet, and the biological and genetic characteristics as well as the pathogenicity in 7-day-old piglets were investigated.

## 2. Materials and Methods

### 2.1. Sample Collection

In January 2017, a farm in Guangxi, China had a serious outbreak of PED, with acute watery diarrhea and vomiting seen in newborn piglets. Small intestine samples were collected from the piglets and were homogenized with 20% glycerin in a PBS stock preservation solution (GPSs). The suspensions were vortexed and centrifuged for 5 min at 3000× *g* at 4 °C. The supernatants were collected and stored at −80 °C until they were utilized.

### 2.2. PEDV Diagnosis and ORF3 Amplification

Total RNA was extracted using a viral DNA/RNA kit (Axygen Scientific, Union City, CA, USA) and transcribed into cDNA using an Oligo dTs primer, dNTP mix and an M-MLV reverse transcriptase reagent (TaKaRa, Dalian, China). For PEDV detection and ORF3 amplification, the primers against N gene (NF, 5′-GAAATAACCAGGGTCGTGGA-3′ and NR, 5′-GCTCACGAACAGCCACATTA-3′) and ORF3 gene (ORF3F, 5′-GTCCTAGACTTCAACCTTACGAAG-3′ and ORF3R, 5′-AACTACTAGACCATTATCATTCAC-3′) were used [20], respectively. The products were purified and cloned into a pMD-18T vector (TaKaRa, Dalian, China), and the sequences were determined by the Beijing Genomics Institute (Guangzhou, China).

### 2.3. PEDV Isolation and Purification

Vero cells were used for PEDV isolation and purification. Vero cells were maintained in high glucose DMEM (Life Technologies, Carlsbad, CA, USA) supplemented with antibiotics and 10% fetal bovine serum (Biological Industries, Kibbutz Beit Haemek, Israel) at 37 °C in a 5% CO_2_ incubator. The supernatants were filtered through a 0.22-μm-pore-size syringe filter. After 1 h of incubation, the cells were maintained in DMEM containing 15 μg/mL trypsin at 37 °C in a 5% CO_2_ atmosphere until a cytopathic effect became visible. The infected cells were lysed using a freeze-thaw method and subsequently centrifuged at 3000× *g* for 5 min at 4 °C.

Vero cells in six-well plates were inoculated with 200 μL of 10-fold serially-diluted PEDV. After 1 h of adsorption at 37 °C, the cell monolayers were washed with phosphate-buffered saline (PBS) and overlaid with 1% low melting point agarose with 15 μg/mL trypsin. After the gel overlay solidified, the plates were inverted and placed in an incubator at 37 °C with 5% CO_2_. At 3–4 days post-infection (dpi), plaques were selected for cell infections or were visualized using crystal violet staining. Two PEDV isolates—one variant bearing a naturally occurring truncated ORF3 gene named 17GXCZ-1ORF3d and another strain possessing a complete ORF3 gene named 17GXCZ-1ORF3c—were plaque-purified serially three times. Ten plaques were randomly selected, and the diameter was measured using a ruler in ImageJ 1.8.0 software. The purified viruses were propagated and the RNA was extracted for the amplification of the ORF3 in order to verify the size of the purified virus.

### 2.4. Indirect Immunofluorescence Assay

Vero cells were grown to 70–80% confluence on 96-well plates and inoculated with the PEDV strains 17GXCZ-1ORF3c and 17GXCZ-1ORF3d, respectively. Medium alone was used as control. At 72 h post-infection (hpi), the cells were fixed with cold formaldehyde and blocked with PBS containing 1% bovine serum albumin (BSA). After blocking, the cells were incubated with an anti-PEDV spike protein monoclonal antibody (Median, Chuncheon, Korea; diluted 1:500) for 2 h at 37 °C. The cells were washed with PBS three times and then incubated with an Alexa FluorTM 488 conjugated goat anti-mouse IgG (H+L) antibody (Invitrogen, CA, MSA; diluted 1:4000) for 1h. Finally, the cells were washed and then visualized using a fluorescent microscopy.

### 2.5. Multi-Step Growth Curves of Viruses

For the growth curve analysis, Vero cells in 12-well plates were inoculated with the PEDV strains 17GXCZ-1ORF3d and 17GXCZ-1ORF3c, respectively, at a multiplicity of infection (MOI) of 0.001. After adsorption at 37 °C for 1 h, followed by three washes with PBS, the supernatants and the infected cells were collected at 6, 12, 24, 36, 48, 60 and 72 hpi and stored at −80 °C for virus titration. The virus titers in Vero cells at each time point were determined in triplicate by using plaque assays.

### 2.6. Whole Genome Sequencing of PEDV

To further characterize the PEDV detected by the ORF3 sequencing, both strains of the plaque-purified viruses were subjected to next-generation sequencing. Libraries were constructed using the TruSeq Stranded mRNA LT Sample Prep Kit (Illumina, San Diego, CA, USA), and these were sequenced on an Illumina HiSeq X Ten platform and 150 bp paired-end reads were generated. The libraries and sequencing were conducted by OE Biotech Co., Ltd. (Shanghai, China). In order to verify the sequences, a total of 27 overlapping fragments of PEDV were amplified using 2 × Taq Plus Master Mix II (Dye Plus) (Vazyme, Nanjing, China) as previously described [26], with a few modifications. The PCR products were gel purified and cloned into a pMD-18T vector (TaKaRa, Dalian, China) and the sequences were determined by the Beijing Genomics Institute (Guangzhou, China).

### 2.7. Multiple Alignments and Phylogenetic Analyses

Seventy-seven reference PEDV strains containing full-length genomes were selected and used for sequence alignments and phylogenetic analyses (Supplementary Table S1). Multiple sequence alignments were generated by applying the MegAlign program. Phylogenetic trees were constructed from the aligned nucleotide or amino acid sequences by using the neighbor-joining method in MEGA5.2 software and subsequently subjected to a bootstrap analysis with 1000 replicates to determine the percentage reliability values of each internal node of the tree. The resulting tree was visualized using iTOLv.5 (Interactive Tree of Life, http://itol.embl.de/, accessed on 6 September 2020).

### 2.8. Experimental Design of Infection

Twelve 7-day-old conventional piglets were purchased from a commercial pig farm with no PEDV vaccination program and no history of PED. All animals were diagnosed as negative for PEDV, transmissible gastroenteritis virus (TGEV), rotavirus (PoRV), porcine deltacoronavirus (PDCoV), classical swine fever virus (CSFV), porcine reproductive and respiratory syndrome virus (PRRSV) and pseudorabies virus (PRV) by a virus-specific RT-PCR analysis of rectal swabs and determined to be free of antibodies to PEDV by a commercial PEDV antibody ELISA kit (Biovet Inc., St-Hyacinthe, QC, Canada). The piglets were randomly assigned into three experimental groups: the 17GXCZ-1ORF3d-inoculated group (*n* = 4), the 17GXCZ-1ORF3c-inoculated group (*n* = 4) and the control group (*n* = 4). The piglets were housed in separate rooms and were artificially fed milk replacer every 4–6 h. The piglets in the infected groups were inoculated orally with 6 log10 PFU/mL (2 mL/piglet) of the PEDV strains 17GXCZ-1ORF3d or 17GXCZ-1ORF3c, respectively, while the uninfected group were treated with the same amount of cell culture media and used as controls. During the experiment, the piglets had their body temperatures and weights measured regularly and were monitored for clinical signs of disease including diarrhea and vomiting. Rectal swabs were collected at different timepoints after PEDV infection for scoring fecal denseness (scores: 0, normal; 1, pasty stool; 2, semiliquid diarrhea; and 3, liquid diarrhea) and placed in EP tubes containing 4 mL of GPSs for the enumeration of fecal viral RNA shedding by RT-quantitative PCR (RT-qPCR). The sequences of primers used were as follows: qMF, 5′-GGAATTTCACATGGAATATCA-3′; and qMR, 5′-CCATAGAATAGCCATCTTGAC-3′. RNA copies were calculated using a generated standard curve.

The animal care and procedures used in this study were handled strictly according to the Animal Care & Welfare of Guangxi University (No: GXU2020-022). Veterinary personnel were involved throughout the study and all necessary safeguards were followed so as to ensure the minimal suffering of animals. In addition, the piglets that approached humane endpoints were euthanized under deep anesthesia with a xylazine combo (0.1–0.2 mL/kg body weight) when necessary. The humane endpoints in this study were a combination of lethargy, anorexia (weight loss > 15% of body weight at infection) and/or if malaise and dehydration resulted in the piglet being unable to stand unaided. However, all surviving piglets from the challenged and control groups were euthanized at 5 dpi for post-mortem examinations. At necropsy, tissue samples including duodenum, jejunum, ileum, cecum, mesenteric lymph nodes (MLN) and stomach were collected and homogenized with GPSs to a final concentration of 0.1 g/mL using a high-throughput tissue grinder (Ningbo Techtronic Biotechnology Co., Ningbo, China). In addition, 3–4 cm each of duodenum, jejunum, ileum, cecum, MLNs and stomach were taken and processed for hematoxylin and eosin (H&E) staining and immunohistochemistry (IHC), respectively. The ORF3 genes from the small intestines of piglets were amplified and sequenced in order to verify which virus they were inoculated with.

### 2.9. H&E and IHC Staining

Tissues of duodenum, jejunum, ileum, cecum, MLNs and stomach were fixed in 4% paraformaldehyde for H&E staining. For each small intestinal section, five villi and crypts were measured using Image-pro plus 6.0 software (Media Cybernetics, Rockville, MD, USA). The villus height versus crypt depth (VH:CD) ratios were calculated as previously described [6]. A monoclonal antibody for the PEDV spike protein (Median, Chuncheon, Korea; diluted 1:500) and HRP conjugated goat anti-mouse IgG (H+L) (Servicebio, Wuhan, China; diluted 1:200) were used for IHC staining, and the results were observed by microscopy (NIKON Eclipse Ci, Japan) and photographed by an imaging system (NIKON digital sight DS-FI2, Japan). The presence of the PEDV antigen was assessed by using a semi-quantitative analysis of tissue sections (−, no cells showed staining; +, ++, +++ represent 1–30%, 30–60% and 60–100% of epithelial cells that showed staining, respectively).

### 2.10. Statistical Analysis

All the values are expressed as the means ± standard error of the means (SEMs). The statistical analysis was performed by a Student’s *t*-test using GraphPad Prism 8 (GraphPad, La Jolla, CA, USA) and a one-way or two-way ANOVA test using SPSS version 25.0 (IBM, Chicago, IL, USA). A value of *p* < 0.05 was considered statistically significant and a value of *p* < 0.01 was considered extremely significant.

## 3. Results

### 3.1. Virus Isolation and Biological Characteristics of 17GXCZ-1ORF3d and 17GXCZ-1ORF3c Strains In Vitro

The results of RT-PCR revealed that the sample was positive for PEDV, and two ORF3 genotypes were present in the same piglet. Since the deletion could have occurred in the cell culture as was described before [27], direct sequencing was conducted to confirm the presence of both variants in the same sample of piglet.

**Figure 1:**
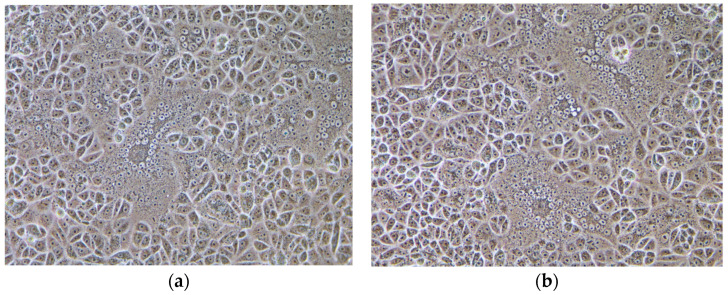

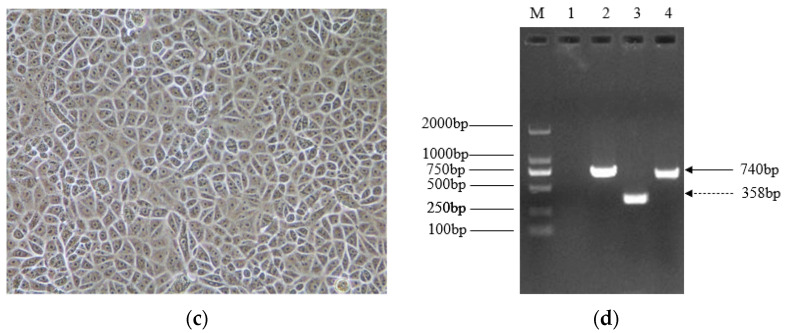
Virus isolation and purification of the PEDV strains with different ORF3 genotypes. CPE formation in Vero cells infected with the PEDV strains of (**a**) 17GXCZ-1ORF3d or (**b**) 17GXCZ-1ORF3c showing rounded and clustered at 48 hpi (200×). (**c**) Vero cells control (200×). (**d**) Detection and amplification of the ORF3 gene in PEDV strains. M: DL 2000 marker; Lane 1: negative control; Lane 2: 17GXCZ-1ORF3c (~740 bp); Lane 3: 17GXCZ-1ORF3d with a large genomic deletion (~358 bp); Lane 4: CV777 strain (~740 bp). The solid arrows indicate the predicted products (740 bp) and the dashed arrows indicate the products of the PEDV variants with a large genomic deletion (~358 bp).

The 17GXCZ-1ORF3d strain bearing a naturally occurring truncated ORF3 gene and the 17GXCZ-1ORF3c strain possessing a complete ORF3 gene were serially plaque-purified three times, respectively. Vero cells infected with the PEDV strains of 17GXCZ-1ORF3d and 17GXCZ-1ORF3c both produced obvious classical cytopathic effects (CPE) including cell fusion, multinucleated giant cell formation and cell detachment (Figure 1a,b). The 17GXCZ-1ORF3d and 17GXCZ-1ORF3c strains were titrated to titers of 7 log10 and 6.26 log10 PFU/mL, respectively. The ORF3 genes of the isolated and purified strains were identified using RT-PCR (Figure 1d). Virus propagation was confirmed by the detection of PEDV antigens with IFA using an anti-PEDV S protein monoclonal antibody. The results revealed that the attached fluorophores could be detected in infected Vero cells, whereas none were detected in the control group.

**Figure 2:**
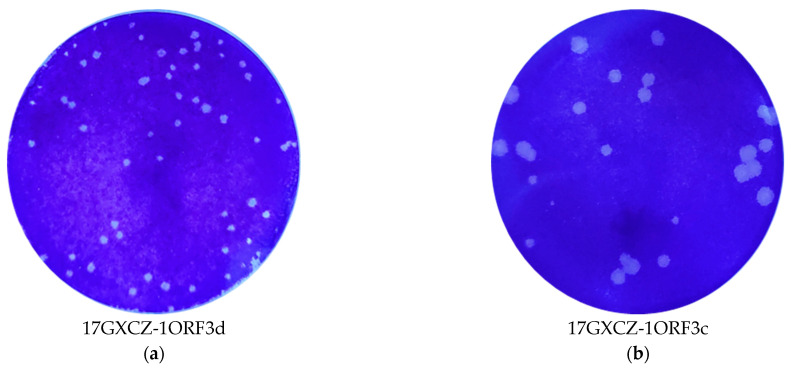

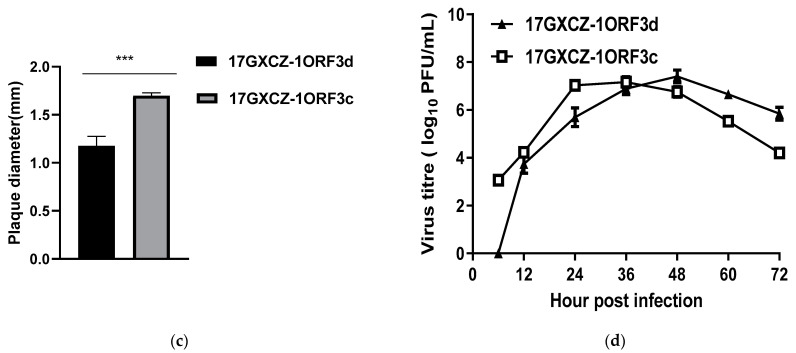
Plaque purification and viral growth kinetics of the PEDV strains 17GXCZ-1ORF3d and 17GXCZ-1ORF3c. Plaque purification of the PEDV strains (**a**) 17GXCZ-1ORF3d and (**b**) 17GXCZ-1ORF3c. Vero cell monolayers were inoculated with 10-fold serially diluted PEDV strains of 17GXCZ-1ORF3d and 17GXCZ-1ORF3c, respectively. After incubation for 1 h, the cells were overlaid with 1% agarose. Plaques were stained with crystal violet at 3–4 dpi. (**c**) The plaque diameters of 17GXCZ-1ORF3d and 17GXCZ-1ORF3c. The diameters of ten randomly selected plaques were measure using a ruler in the ImageJ 1.8.0 software. *** represents that the mean diameters of plaques formed by 17GXCZ-1ORF3d (1.18 mm) were significantly smaller than those of 17GXCZ-1ORF3c (1.70 mm), with *p* values of <0.001. (**d**) Viral growth kinetics of 17GXCZ-1ORF3d and 17GXCZ-1ORF3c in Vero cells. Vero cells were inoculated with the 17GXCZ-1ORF3d and 17GXCZ-1ORF3c strains at MOI = 0.001, respectively. The infected cells were collected at 6, 12, 24, 48, 60 and 72 hpi for virus titrations. The virus titers at each time point were determined in triplicate using plaque assays.

We compared the plaque sizes and morphology between the 17GXCZ-1ORF3d and 17GXCZ-1ORF3c PEDV strains. The mean diameter of the plaques formed by 17GXCZ-1ORF3d (1.18 mm) was significantly smaller than that observed for 17GXCZ-1ORF3c (1.70 mm) (Figure 2c). The growth properties of the two plaque-purified PEDV isolates were investigated by the multi-step growth assay with an MOI of 0.001. As shown in Figure 2d, the 17GXCZ-1ORF3d strain exhibited growth kinetics that were not exactly similar to 17GXCZ-1ORF3c. 17GXCZ-1ORF3d reached a peak with a mean titer of 7.4 log10 PFU/mL at 48 hpi, while 17GXCZ-1ORF3c reached a peak mean titer of 7.16 log10 PFU/mL at 36 hpi.

### 3.2. Full-Length Genome Sequence Analysis and Phylogenetic Characterization of PEDV 17GXCZ-1ORF3d and 17GXCZ-1ORF3c Strains

We determined the full-length genomes of 17GXCZ-1ORF3d (GenBank accession no. MT547179) and 17GXCZ-1ORF3c (GenBank accession no. MT547180) to be 27662 and 28,044 nucleotides (nt), respectively. We compared these genomes with 77 representative strains listed in the GenBank and performed further analysis using the Clustal W method with the MegAlign program and MEGA5.2 software. 17GXCZ-1ORF3c had a complete ORF3 gene sequence, with a length of 675 bp, and encoded a protein of 224 amino acids (aa), whereas the ORF3 gene of 17GXCZ-1ORF3d was 293 bp in length, containing a continuous deletion from 172–554 bp, and encoded a truncated protein of 89aa. 17GXCZ-1ORF3d had >99% nt identity with 17GXCZ-1ORF3c in the 5′UTR, ORF1a/1b, S, E, M, N and 3′UTR regions but demonstrated much lower nt identities (80.5%) in the ORF3 gene (Table 1). The whole genomes of 17GXCZ-1ORF3d and 17GXCZ-1ORF3c shared 98.3% nucleotide identity with the group G2 prototype strain, AJ1102, but had 96.7% identity with the subgroup G1 prototype strain, CV777. Compared to the ORF3 gene of all the representative strains, 17GXCZ-1ORF3c showed 90.9–99.3% nt and 91.3–100% aa identities, whereas the nt and aa identities of 17GXCZ-1ORF3d were lower at 76.7–84.8% and 73.0–83.3%, respectively.

**Figure 3:**
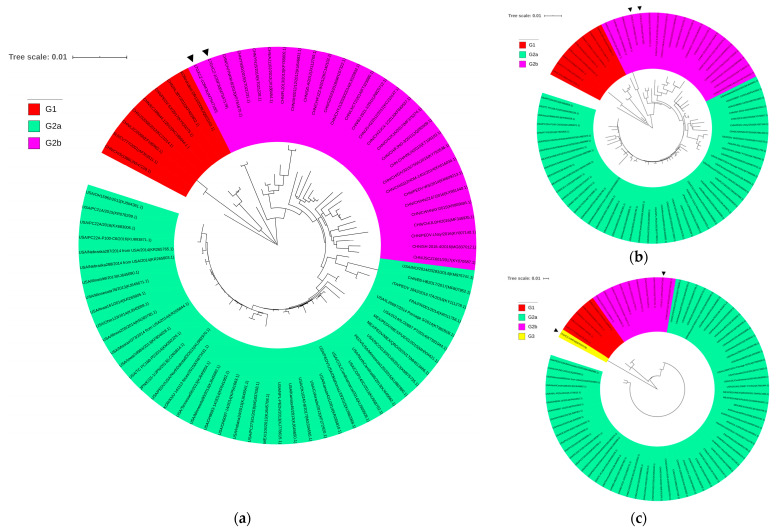
Genotyping and origin of the PEDV strains 17GXCZ-1ORF3d and 17GXCZ-1ORF3c based on different genes. Phylogenetic trees were constructed based on the aligned full-length genomes (**a**) S protein and (**b**) ORF3 protein (**c**) by using the neighbor-joining method from MEGA 5.2, with 1000 bootstrap replicates. Scale bars represent the branch lengths measured by the number of substitutions per site. Each PEDV strain is indicated in the following format: country of origin (three letter code: CHN, China; JPN, Japan; KOR, Korea; MEX, Mexico; SUI, Switzerland; and USA, the United States)/strain name/year of sample collection (Genbank accession number). Group 1, G2a subgroup, G2b subgroup and Group 3 are coded in red, green, pink and yellow, respectively. The triangle symbols represent the strains obtained in this study.

To investigate the evolution of PEDV, we further constructed phylogenetic trees based on the S and ORF3 proteins as well as on the whole genome sequences (Figure 3). Both 17GXCZ-1ORF3d and 17GXCZ-1ORF3c had evolved into subgroup G2b based on their whole genome and S protein sequences. The strains of 17GXCZ-1ORF3d and 17GXCZ-1ORF3c formed an independent clade within the same subgroup, which clustered closely around the Chinese isolates such as the AJ1102 strain, but were distant from the North America epidemic strains. The phylogenetic analysis based on the ORF3 proteins of the 17GXCZ-1ORF3c strain was divided into subgroup G2b, but 17GXCZ-1ORF3d clustered into a new G3 group. The ORF3 gene-based phylogenetic tree suggested that 17GXCZ-1ORF3d was a novel strain with a naturally occurring large deletion at the 172–554 bp position in the ORF3 gene.

### 3.3. Pathogenicity of 17GXCZ-1ORF3d and 17GXCZ-1ORF3c Strains in Piglets

**Figure 4:**
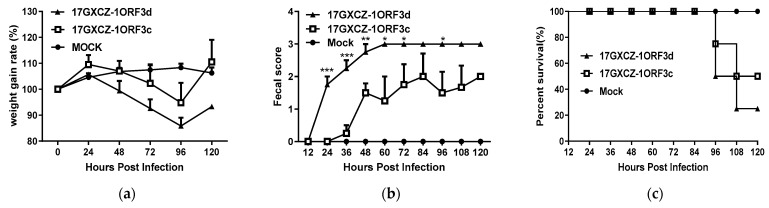

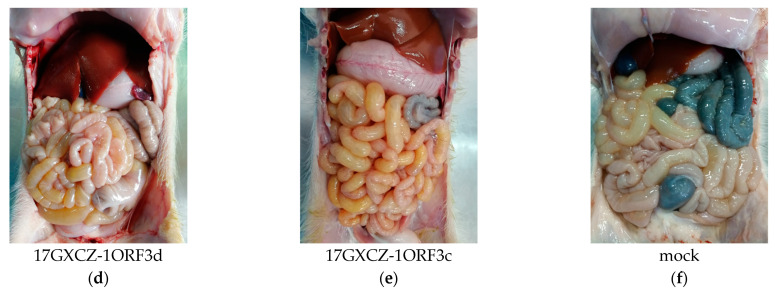
Pathogenicity analysis of the PEDV strains 17GXCZ-1ORF3d and 17GXCZ-1ORF3c. (**a**) The average body weight changes in each group. (**b**) Fecal scores of piglets in different groups. Rectal swabs were collected at different timepoints after PEDV infection, scores standard: 0, normal; 1, pasty stool; 2, semiliquid diarrhea; and 3, liquid diarrhea. (**c**) The survival rate of piglets in each group. After oral inoculation with PEDV, one piglet died at 96 hpi and one at 108 hpi in the 17GXCZ-1ORF3c group, while two piglets died at 96 hpi and one piglet died at 108 hpi in the 17GXCZ-1ORF3d group. Each data point in the graph represents the average value from four or all of the surviving animals ± SEM. The asterisks indicate significant differences between the groups of 17GXCZ-1ORF3d and 17GXCZ-1ORF3c (* *p* < 0.05; ** *p* < 0. 01; *** *p* < 0 .001). Gross lesions found in piglets of (**d**) 17GXCZ-1ORF3d, (**e**) 17GXCZ-1ORF3c and (**f**) mock groups at necropsy. The piglets were necropsied upon death, whereas all surviving piglets from the challenged and mock groups were euthanized at 5 dpi for post-mortem examinations.

The pathogenicity characteristics of the PEDV strains of 17GXCZ-1ORF3d and 17GXCZ-1ORF3c were compared. Twelve 7-day-old piglets were randomly divided into three groups and were orally inoculated with 17GXCZ-1ORF3c and 17GXCZ-1ORF3d at a dose of 6 log10 PFU/mL (2 mL/piglet) and DMEM (2 mL/piglet), respectively. During the challenge period, the piglets in the infected groups exhibited severe diarrhea, vomiting and weight loss (Figure 4a). The piglets in the 17GXCZ-1ORF3c group began to show diarrhea at 36 hpi, while piglets in the 17GXCZ-1ORF3d group developed diarrhea at 24 hpi, which was 12 h earlier than the piglets in the former group. Notably, the fecal scores of the 17GXCZ-1ORF3d group were significantly higher than those of the 17GXCZ-1ORF3c group throughout most of the duration of the experiment (Figure 4b).

In this study, the mortalities of the 17GXCZ-1ORF3d and 17GXCZ-1ORF3c strains reached 75% (3/4) and 50% (2/4), respectively (Table 2). After the oral inoculation with PEDV, one piglet died at 96 hpi and one at 108 hpi in the 17GXCZ-1ORF3c group, while two piglets died at 96 hpi and one piglet died at 108 hpi in the 17GXCZ-1ORF3d group. (Figure 4c). The results of the pathological examination revealed that the intestinal walls of the piglets in both the 17GXCZ-1ORF3d and 17GXCZ-1ORF3c groups were thin or even transparent, with a large amount of yellowish fluid in the intestinal cavities (Figure 4d,e). No piglets died or exhibited any clinical signs in the control group (Figure 4f).

The viral shedding of feces was investigated by RT-qPCR. The fecal shedding occurred at 36 hpi from the piglets in the 17GXCZ-1ORF3c group, whereas the earliest detection of fecal virus shedding from the piglets in the 17GXCZ-1ORF3d group was at 24 hpi, which was 12 h earlier than in the 17GXCZ-1ORF3c group. It coincided with the onset of clinical signs. Piglets in the groups of 17GXCZ-1ORF3d and 17GXCZ-1ORF3c shed the virus at the highest level within 96 hpi, with the mean titers of 6.68 log10 copies/mL and 6.43 log10 copies/mL, respectively (Table 2).

The viral loads in different tissues including the duodenum, jejunum, ileum, cecum, MLNs and stomach were determined. As shown in Table 2, the segments of duodenum, jejunum and ileum exhibited high viral loads in both the 17GXCZ-1ORF3d and 17GXCZ-1ORF3c groups ranging from 6.18 to 7.12 log10 copies/g. The viral load in the duodenum and jejunum from the 17GXCZ-1ORF3d group was higher than that of the 17GXCZ-1ORF3c group, but this was not significant. Neither virus shedding in the feces nor the viral load in the tissues of the control group was detected.

**Figure 5:**
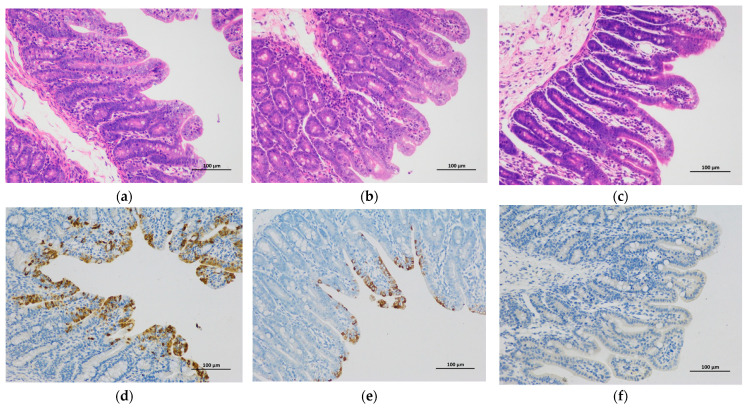
H&E and IHC staining of the jejunum from the PEDV-infected and mock piglets. Different tissue samples including the duodenum, jejunum, ileum, cecum, MLNs and stomach from each group were taken and then processed for H&E and IHC staining, and representative images of the jejunum are shown (200×). H&E staining of the jejunum in piglets from the (**a**) 17GXCZ-1ORF3d, (**b**) 17GXCZ-1ORF3c and (**c**) mock groups. The jejunum from the PEDV-infected piglets was characterized by the shortening, atrophy or even the shedding of intestinal villus. IHC staining of the jejunum from the (**d**) 17GXCZ-1ORF3d, (**e**) 17GXCZ-1ORF3c and (**f**) mock groups. PEDV antigen appears as a brown stain and was detected in the epithelial cells of the jejunum in 17GXCZ-1ORF3d and 17GXCZ-1ORF3c infected piglets. The intestinal tissue sections were stained with an anti-PEDV spike protein monoclonal antibody followed by the incubation with an HRP conjugated goat anti-mouse antibody and then visualized using a fluorescent microscope.

Using H&E staining, the infected piglets were characterized by the shortening, atrophy or even the shedding of intestinal villi (Figure 5). To investigate the severity of atrophic enteritis in infected piglets, the VH:CD ratios of different intestinal segments from the infected piglets were compared with those of the piglets in the mock group (Table 2). Piglets in the 17GXCZ-1ORF3c group had significantly decreased VH:CD ratios in duodenum (2.32 ± 0.08) and ileum (2.42 ± 0.12) compared with the piglets in the control group. In addition, in the 17GXCZ-1ORF3d group, the VH:CD ratios in the duodenum (2.38 ± 0.15) and jejunal (1.94 ± 0.17) were significantly lower than those in the mock group (*p* < 0.05), and the VH:CD ratios in the ileum (1.96 ± 0.16) were markedly lower than those in the mock group (*p* < 0.01).

After the euthanasia of piglets, PEDV-specific IHC staining was performed on serial sections of the duodenum, jejunum, ileum, cecum, MLNs and stomach of all groups (Figure 5d,f). The cecum and stomach had almost no PEDV antigen signal, and MLNs in the infected piglets were slightly enlarged and contained a few of PEDV antigen signals (data not shown). PEDV antigen positive signals were observed in the duodenum (30–60%), jejunum (60–100%) and ileum (1–30%) in the 17GXCZ-1ORF3d group (Table 2). The PEDV antigen signal in the villous enterocytes of the duodenum and jejunum from the 17GXCZ-1ORF3d group was higher than that in those from the 17GXCZ-1ORF3c group. None of the four piglets in the control group was IHC positive.

## 4. Discussion

Coronaviruses infect humans and various animal species, causing respiratory, gastrointestinal and neurological diseases. PEDV belongs to a group of enteropathogenic swine coronaviruses of the genus alphacoronavirus, causing large economic losses to the global swine industry [2]. Novel PEDV strains with large deletions in the S gene have been found in different countries [20]. Based on the S gene, PEDVs can naturally coexist with multiple genotypes and can cause new tropisms [21]. There were three ORF3 genotypes reported in China as early as 2010 [18]. The ORF3 protein of the HLJBY strain only consists of 91 aa, with 133 aa deletions at the C’ end [24]. Recently, we discovered a new ORF3 genotype that encoded a truncated protein of 89 aa, with continuous deletions from 172–554 bp [25]. However, no co-infection of multiple ORF3 genotypes has been reported.

Based on the complete genome sequences, PEDV evolved into two distinct groups, G1 (classical) and G2 (variant), and most of the prevailing strains since 2010 have been grouped into the G2 group. Geno-group G2 was further subdivided into G2a and G2b subgroups in this study, and multiple recombinants and the S-INDEL strains worldwide could be clustered into a new G2c subgroup by the other proposed systems [13,28]. The S protein, which was expected to be the primary determinant of attenuation, contains two subunits, S1 and S2, responsible for receptor binding and membrane fusion, respectively [29]. In addition, there are at least four neutralizing epitopes of PEDV that have been identified in the S protein, including COE (499–638), SS2 (748–755), SS6 (764–771) and 2C10 (1368–1374) [1]. In this study, there are four amino acid differences (G888R, O1009N, S1064C and G1068D) in the S protein between the PEDV strains 17GXCZ-1ORF3d and 17GXCZ-1ORF3c. Previous studies have shown that the amino acid position 888 is in close proximity to the S2′ cleavage site, and it may play an important role for the viability and the cell fusion capacity of the virus [30,31]. However, whether these mutations will affect the plaque size, growth kinetics or virulence of the virus warrants further investigation.

The ORF3 gene of coronaviruses carry accessory genes known to be associated with viral virulence [16], replication [32] and adaptation [14,17]. The ORF3 is the only accessory gene in PEDV, and it influences virus production and virulence [16,33]. The PEDV ORF3 can delay the S phase in dividing cells and suppress cell cycle progression, and it causes endoplasmic reticulum stress in order to facilitate autophagy [34]. PEDV ORF3 protein promotes virus proliferation by inhibiting cell apoptosis caused by viral infections [15]. Moreover, PEDV ORF3 is involved in the innate host immune response, where it has been found to inhibit the IFN-β and IRF3 promoter activities [35] as well as significantly inhibit the production of pro-inflammatory cytokines such as interleukin-6 (IL-6) and IL-8 [36]. 17GXCZ-1ORF3d is a novel strain with a naturally occurring large deletion at the 172–554 bp position in the ORF3 gene, and the functional effects of this truncated ORF3 gene in the activity of the virus need to be further investigated.

The 17GXCZ-1ORF3d strain generated significantly smaller plaques and had slightly lower growth kinetics when compared to the 17GXCZ-1ORF3c strain in the present study. It was previously demonstrated that a recombinant infectious clone derived PEDV, in which the ORF3 was replaced with a red fluorescent protein, produced a reduced plaque size compared to the parental strain [37]. These results indicated that the ORF3 might be important for plaque formation and viral growth in vitro. Recent studies revealed that a highly pathogenic PEDV rBJ2011C strain made smaller plaques and had slower growth kinetics than a low virulent rCHM2013 strain [26]. The pathogenicity of PEDV was associated with the virus strain, but the impact of other factors, such as plaque formation and virus growth kinetics in vitro, requires further investigation.

Nursing piglets (<10-day-old) infected with PEDV began to show diarrhea at 24 hpi or several hours later, and this mostly coincided with the first detection of viral shedding in the feces [38,39]. In agreement with the above results, the onset of diarrhea was 12 h earlier and the first detection of fecal virus shedding was 24 h earlier in the piglets inoculated with the 17GXCZ-1ORF3d strain compared to those in the 17GXCZ-1ORF3c group, indicating a shorter incubation time for the former strain. The fecal shedding of the viruses in both groups peaked at 96 hpi and the titers decreased markedly during the recovery stage of the infection by 120 hpi, and this may be possibly due to PED re-infection of regenerating enterocytes. The piglets died with severe diarrhea [40].

The VH:CD ratios were reduced along with a marked shortening of the villi following the PEDV infection [2], and the jejunum and ileum may be the primary sites during acute PEDV infection in nursing pigs [41]. In our study, the VH:CD ratios in all the small intestinal segments from the 17GXCZ-1ORF3d group were significantly lower than those in the control group. Moreover, compared with the 17GXCZ-1ORF3c group, the animals in the 17GXCZ-1ORF3d group had higher but not significant VH:CD ratios in the jejunum and ileum, viral load in all small intestinal segments and PEDV antigen signals in the villous enterocytes of the duodenum and jejunum. These observations indicated that the PEDV 17GXCZ-1ORF3d strain causes serious damage to the villous epithelial cells and may have a similar viral replication ability in vivo.

Generally, the naturally truncated form of ORF3 might cause the attenuation of PEDV in a natural host [18,24], but not all ORF3 truncations have been associated with reduced pathogenicity [42]. The virulence of the PEDV P55 strain with a truncated ORF3 did not differ from that of the other field isolates with full-length ORF3 [43]. The cell-adapted PEDV YN15 strain gained an early termination of the ORF3 gene at 145 aa, and the results of clinical symptom analyses, necropsies and IHC from pig infection experiments showed that this is a virulent PEDV strain [44]. Here we report a PEDV strain, 17GXCZ-1ORF3d, which has a naturally truncated ORF3 gene containing a continuous 382 nucleotides deletion from 172–554 bp and has been verified as a virulent strain causing severe diarrhea and high mortality in suckling piglets. So far, there is conflicting data regarding the role of ORF3 and its importance for viral pathogenicity. The striking sequence differences between the PEDV strains 17GXCZ-1ORF3d and 17GXCZ-1ORF3c are located in the ORF3 gene, particularly at the 172–554 bp position of the ORF3 gene. Previous studies have demonstrated that the naturally-occurring amino acid deletion could abolish the suppressive effect of some ORF3 variants on virus replication in vitro [45]. The naturally truncated ORF3 protein displayed a more apparent association with the S protein, and it may work in concert to regulate PEDV replication in vivo [14]. The sequence difference in the ORF3 gene may be responsible for the pathogenicity differences between these two PEDV strains, but this remains to be confirmed in further studies.

Currently, the distribution of PEDV with a naturally truncated ORF3 gene in China or globally is unknown. Apart from coinfection with the intact ORF3 gene strain, whether the naturally truncated ORF3 strain will infect the piglets alone in the field or even cause a larger outbreak has to be carefully monitored. In this study, the piglets infected with the PEDV strains of 17GXCZ-1ORF3d and 17GXCZ-1ORF3c exhibited severe clinical signs. However, there is a need to increase the animal number to strengthen statistical power when comparing the pathogenicity between these two strains, and the correlation between the naturally truncated ORF3 gene and pathogenicity needs to be confirmed by reverse genetics.

## 5. Conclusions

In conclusion, we identified two PEDV strains with different ORF3 variants from the same pig, which illustrates the complexity involved in the clinical prevention and control of PED. PEDV strains with a large deletion in the ORF3 gene might have the advantage of acting as a genetic marker for potential vaccine development.

## Figures and Tables

**Table 1:** Comparisons of the nucleotide sequences of the genome organization of the 2017 Guangxi isolates and their genogroup representative PEDV strains.

Gene	17GXCZ-1ORF3d (MT547179)		17GXCZ-1ORF3c (MT547180)		CV777 (AF353511)		AJ1102 (JX188454)	
	Size (nt)	Size (aa)	Size (nt)	Identity (%)	Size (nt)	Identity (%)	Size (nt)	Identity (%)
5′UTR	292	/	292	100	296	97.3	292	98.6
ORF1a/1b	20,345	6781	20,345	100	20,345	97.2	20,344	98.4
S	4158	1386	4158	99.9	4152	94.1	4158	97.9
ORF3	293	89	675	80.5	675	78.1	675	79.5
E	231	76	231	99.1	231	97.4	231	99.6
M	681	226	681	99.7	681	98.4	681	99.4
N	1326	441	1326	100	1326	95.9	1326	97.7
3′UTR	334	/	334	100	334	95.8	343	98.2
Total	27,662	/	28,044	99.9	28,033	96.7	28,044	98.3

**Table 2:** Pathogenicity analysis of the PEDV strains 17GXCZ-1 ORF3d and 17GXCZ-1 ORF3c found in China, 2017 †.

Groups	Inoculum Dose (2 mL)	Mortality	Fecal Shedding, Log10 Copies/mL, by PIH, Mean						Onset of Clinical Signs as Judged by PIH	Quantification of Viral Load, Log10 Copies/mL, Mean			VH: CD, Mean			PEDV Antigen Detection in Frozen Tissues ‡		
			0	24	48	72	96	120		D	J	I	D	J	I	D	J	I
Mock	Cell culture media	0%, 4/4	-	-	-	-	-	-	-	-	-	-	3.32	2.44	2.86	-	-	-
17GXCZ-1 ORF3d	6 log10 PFU/mL	75%, 1/4	-	1.72	5.89	6.43	6.68	4.66	24-48	6.32	7.12	6.90	2.38 *	1.94 *	1.96 **	++	+++	+
17GXCZ-1 ORF3c	6 log10 PFU/mL	50%, 2/4	-	-	4.94	5.76	6.43	3.46	36-48	6.18	6.42	6.87	2.32 *	2.00	2.42 *	+	+	++

† PEDV, porcine epidemic diarrhea virus; PIH, post inoculation hour; VH:CD, ratio of villous height to crypt depth; D-duodenum, J-jejunum, I-ileum; PFU, plaque formation unit; -, no result. ‡ PEDV antigen detection in frozen tissues. +, ++, and +++ denotes less than 30%, 30–60% and more than 60% of villous enterocytes showing a PEDV antigen positive signal, respectively. Viral load in the fecal wastes and in the tissues of the small intestine was detected by RT-quantitative PCR (RT-qPCR). * and ** represent significant differences when compared to the mock group, with *p* values of <0.05 and <0.01, respectively.

## Data Availability

The complete genome sequences of the PEDV strains 17GXCZ-1ORF3d and 17GXCZ-1ORF3c obtained in this study have been deposited in the GenBank under the accession number MT547179 and MT547180, respectively.

## Supplementary Materials

The following are available online at https://www.mdpi.com/article/10.3390/v13081562/s1, Table S1: Information regarding the reference strains of PEDV.
