# Supplementary material for: Comparative Characterization and Pathogenicity of a Novel Porcine Epidemic Diarrhea Virus (PEDV) with a Naturally Occurring Truncated ORF3 Gene Coinfected with PEDVs Possessing an Intact ORF3 Gene in Piglets
Source: Viruses. 2021 Aug 7;13(8):1562. doi: 10.3390/v13081562 (PMC8402686; doi:10.3390/v13081562)
Supplement: Supplementary file 1 [file viruses-13-01562-s001.zip › viruses-1328270-supplementary.pdf]

Supplementary Table S1. Information regarding the reference strains of PEDV.

Virus strain	Country of origin	Collection date	Accession number
CH/S	China	1986	JN547228.1
CV777	Switzerland	2001	AF353511.1
LZC	China	2006	EF185992.1
JS2008	China	2008	KC109141.1
virulent DR13	South Korea	2009	JQ023161.1
CH/FJND-3/2011	China	2011	JQ282909.1
AJ1102	China	2011	JX188454.1
BJ-2011-1	China	2011	JN825712.1
AH 2012/12	China	2012	KU646831.1
CH/FJZZ-9/2012	China	2012	KC140102.1
CH/ZJCX-1/2012	China	2012	KF840537.1
GD-A	China	2012	JX112709.1
GDS23	China	2012	MH107322.1
JS-HZ2012	China	2012	KC210147.1
KC189944.1	China	2012	KC189944.1
CHYJ130330	China	2013	KJ020932.1
FL2013	China	2013	KP765609.1
KGS-1/JPN/2013	Japan	2013	LC063814.1
NPL-PEDv/2013	USA	2013	KJ778615.1
PC21A	USA	2013	KR078299.1
PC22A	USA	2013	KX683006.1
USA/Colorado/2013	USA	2013	KF272920.1
USA/NorthCarolina66/2013	USA	2013	KJ645662.1
TC PC168-P2	USA	2013	KM392226.1
USA/Iowa/18984/2013	USA	2013	KF804028.1
CO/P14/IC	USA	2013	KU558702.1
MEX/104/2013	Mexico	2013	KJ645708.1
USA/Indiana34/2013	USA	2013	KJ645641.1
USA/Minnesota76/2013	USA	2013	KJ645671.1
USA/NC/2013/35140	USA	2013	KM975735.1
USA/Tennesse56/2013	USA	2013	KJ645654.1
USA/Minnesota90/2013	USA	2013	KJ645682.1
USA/Illinois98/2013	USA	2013	KJ645690.1
USA/Kansas46/2013	USA	2013	KJ645650.1
OH15962	USA	2013	KJ584361.1
CH/GDZHDM/1401	China	2014	KX016034.1
LNCT2	China	2014	KT323980.1
PEDV-WS	China	2014	KM609213.1
FR/001/2014	France	2014	KR011756.1
KNU-141112-feces	South Korea	2014	KR873431.1

COL/Cundinamarca/2014	USA	2014	KU569509.1
OH8593-14	USA	2014	KP641662.1
USA/2014/IL/20697 P7	USA	2014	KT591944.1
USA/IL20697/2014 Passage 5	USA	2014	KT860508.1
USA/Illinois259/2014	USA	2014	KR265785.1
USA/Iowa161/2014	USA	2014	KR265805.1
USA/Kansas431/2014	USA	2014	KR265819.1
OH9097-14	USA	2014	KP641663.1
USA/Nebraska288/2014 from USA	USA	2014	KR265803.1
USA/Missouri373/2014 from USA	USA	2014	KR265844.1
USA/MO/2014/03293	USA	2014	KM975741.1
USA/Nebraska287/2014	USA	2014	KR265765.1
USA/Ohio123/2014	USA	2014	KJ645699.1
HLJBY	China	2015	KP403802.1
CH/GX/2015/750A	China	2015	KY793536.1
CH/HNAY/2015	China	2015	KR809885.1
CH/HNLH/2015	China	2015	KT199103.1
CH/YNKM-8/2013	China	2015	KF761675.1
YN15	China	2015	KT021228.1
YN90	China	2015	KT021231.1
PC22A-P100-C6	USA	2015	KU893871.1
PEDV/USA/Minnesota125/2015	USA	2015	KU982980.1
PEDV/MEX/PUE/01/2015	Mexico	2015	MH004421.1
PEDV/USA/NorthDakota93/2015	USA	2015	KU982970.1
PEDV/USA/Oklahoma133/2015	USA	2015	KU982968.1
CH/JLDH/2016	China	2016	MF346935.1
CH/HNZZ47/2016	China	2016	KX981440.1
CHN/SH-2016-4/2016	China	2016	MG837012.1
JSCZ1601	China	2016	KY070587.1
PEDV-LNsy	China	2016	KY007140.1
PEDV 1842/2016 ITA	Italy	2016	KY111278.1
B5-HB2017	China	2017	MF807952.1
CH/JXJA/2017	China	2017	MF375374.1
PEDV-SX	China	2017	KY420075.1
PEDV/MEX/QRO/02/2017	Mexico	2017	MH013466.1
PC273/O	USA	2017	MG837058.1
USA/OK10240-8/2017	USA	2017	MG334555.1
